# Anemia in salmon aquaculture: Scotland as a case study

**DOI:** 10.1016/j.aquaculture.2021.737313

**Published:** 2022-01-15

**Authors:** A.R. Currie, D. Cockerill, M. Diez-Padrisa, H. Haining, F.L. Henriquez, B. Quinn

**Affiliations:** a School of Health and Life Sciences, University of the West of Scotland, Paisley, Scotland, UK; b WellFish Diagnostics Ltd, University of the West of Scotland, Paisley, Scotland, UK; c Scottish Salmon Company, 8 Melville Crescent, Edinburgh, Scotland, UK; d Mowi Scotland Ltd, Blar Mhor Industrial Estate, Fort William, Scotland, UK; e School of Veterinary Medicine, University of Glasgow, Glasgow, Scotland, UK

**Keywords:** Atlantic salmon, Aquaculture, Anemia, Hematology

## Abstract

Anemia in salmonid aquaculture is a recognized blood disorder resulting from the reduction of hemoglobin concentration and/or erythrocyte count. Because of sub-optimal oxygen supply to the tissues, as a negative impact of anemia fish will experience reduced growth and poor health. This health challenge may be linked with several factors including anthropogenic changes in the marine environment, infectious etiology (viral, bacterial, and parasitic), nutritional deficiencies, or hemorrhaging. From the mid-late summer of 2017 to 2019, Scottish salmon farming companies began to report the occurrence of anemic events in open-net marine sites. At that time, the industry had little understanding of the pathogenesis and possible mechanisms of anemia and limited the ability to formulate effective mitigation strategies. Clinical examination of fish raised suspicion of anemia and this was confirmed by generating a packed cell volume value by centrifugation of a microhematocrit tube of whole anticoagulated blood. Company health team members, including vets and biologists, reported discoloration of gills and local hemorrhages. This paper reviews various commercially significant cases and lesser-known cases of anemia in cultured salmonid species induced by various biological factors. The current methods available to assess hematology are addressed and some future methods that could be adopted in modern day fish farming are identified. An account of the most recent anemic event in Scottish farmed Atlantic salmon (*Salmo salar*) is presented and discussed as a case study from information provided by two major Scottish salmon producers. The percent of total marine sites (*n* = 80) included in this case study, that reported with suspected or clinical anemia covering the period mid-late summer 2017 to 2019, was between 1 and 13%. The findings from this case study suggest that anemia experienced in most cases was regenerative and most likely linked to blood loss from the gills.

## Highlights

• Anemia outbreaks identified in 1–13% of Scottish Atlantic salmon farms surveyed mid-late summer 2017 to 2019.
• In most cases, anemia was regenerative and likely linked to blood loss via the gills.
• Anemia is most likely associated with microhemorrhages identified on the gills and liver.
• Routine hematology required for fish health monitoring and the development of automated analyzers is required.

## 1 Introduction

Aquaculture is regarded as a vital industry that contributes to food security and safeguards nutritional requirements for an expanding global population predicted to reach 9.8 billion by 2050 (United Nations, 2019). Current trends show that food production from aquaculture continues to increase annually, whilst total capture fisheries have stagnated over the past decade or so, as human consumption and utilization of fish and fish products continues to rise (Food and Agriculture Organisation, 2018). The major Atlantic salmon (*Salmo salar*) producing countries include Norway (55%), Chile (25.5%), Scotland (7.6%), and the remaining 12.6% production spread between ten other countries (Kenyon and Davies, 2018; Iverson et al., 2020). In 2018, the global production of farmed Atlantic salmon had exceeded 2.4 million tonnes equating to a 64% production increase compared to volumes produced in 2009 (Kontali Analyse, 2019). Scotland's salmon sector has been estimated to support close to £2 billion revenue for the UK economy (Marsh, 2019). Total production volumes of Scottish Atlantic salmon have fluctuated over the past two decades, with reductions attributed mostly to biological challenges (Table 1). Farming of Atlantic salmon is a relatively young sector when compared to terrestrial animal farming, although both practices must share fundamental approaches to tackle emerging health challenges inherent in the domestication of any new species, to reduce economic loss, and most importantly, safeguard animal welfare for both ethical and regulatory reasons (OIE, 2014; Raja and Jithendran, 2015).

**Table 1:** Production of Scottish Atlantic salmon from 1999 to 2019 and the projected production in 2020 (denoted by **) as estimated by the industry based on on-grown stocks (adapted from Munro, 2020).

Year	Tonnes	% difference	Year	Tonnes	% difference
1999	126,686	14	2010	154,164	6.9
2000	128,959	2	2011	158,018	2.5
2001	138,519	7	2012	162,223	2.7
2002	144,589	4	2013	163,234	0.6
2003	169,736	17	2014	179,022	9.7
2004	158,099	−7	2015	171,722	−4.1
2005	129,588	−18	2016	162,817	−5.2
2006	131,847	2	2017	189,707	16.5
2007	129,930	−1.4	2018	156,025	−17.8
2008	128,606	−1	2019	203,881	30.7
2009	144,247	12	2020	207,630**	

Various health risks to cultured aquatic livestock have emerged from commercial-scale, open-net marine farming, nevertheless, over the past 40 years, both global and Scottish salmon farming have addressed these challenges and undergone considerable expansion. The continued expansion of the salmon farming sector depends partly upon ongoing innovation in disease management strategies. Improvements in these strategies rely upon advances in understanding existing challenges and the identification and characterization of emerging health issues. Existing health challenges in the Scottish salmon farming sector can be broadly summarized into infectious (parasitic, bacterial, viral, or fungal) and non-infectious (environmental, nutritional, genetic) (Hammell et al., 2009). The greatest threat to salmon health is from parasitic sea lice (*Lepeophtheirus salmonis* and *Caligus* spp*.*) (Costello, 2006, Costello, 2009) and amoebic gill disease (AGD) (*Neoparamoeda perurans*) (Rodger, 2013; Hvas et al., 2017). These parasitic infections caused significant health and welfare implications and economic losses through the requirement for treatment interventions, and the potential for poor growth and mortalities if left untreated (Rae, 2002; Costello, 2006, Costello, 2009; Lees et al., 2008).

An emerging health challenge for farmed Atlantic salmon has been the occurrence of anemia during the marine growing phase, although there is very limited information regarding the etiology. In general, anemia can be associated with many of the most commercially significant infectious and non-infectious diseases and is relatively common in various species of intensely farmed livestock e.g., ruminants (Katsogiannou et al., 2018), sheep and goats (Neimark et al., 2004), chicken (Simeonov et al., 2014). The full application of hematology for fish faces challenges due to the genetic and physiological variation seen in the blood of fish, which can be identified between species and even within species. Hematology values can be affected significantly by intrinsic (e.g., age, sex) and extrinsic factors (e.g., temperature, photoperiod) as well as preanalytical variables (e.g., sample time to analysis, anticoagulant) (see review by Fazio, 2019). Thus, for emerging hematological disorders, it can be difficult to rapidly isolate the source of the syndrome to prevent reoccurrence for future generations.

**Fig. 1:**
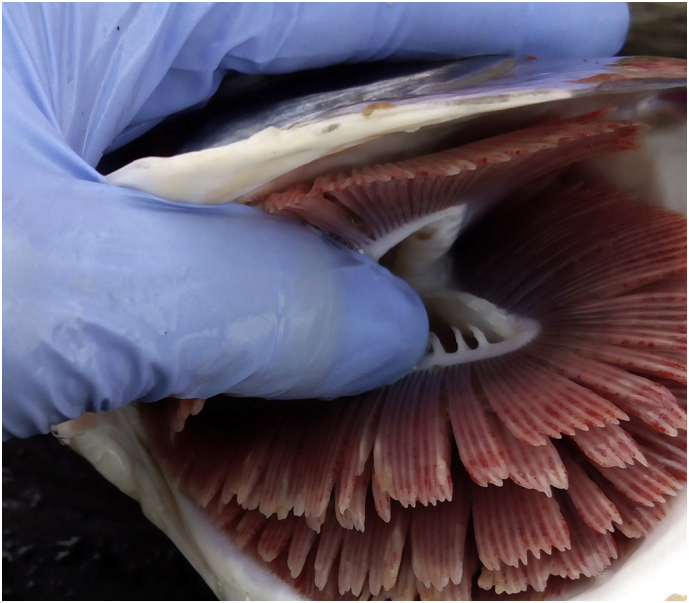
Photo of the gills of a farmed Atlantic salmon (*Salmo salar*) with confirmed anemia. The gill filaments are pale and exhibit pinhead hemorrhages/ microhemorrhages.

Anemia is defined as a reduction in the number of red blood cells and/or the hemoglobin (Hb) concentration of the blood below a normal level (Hoffbrand et al., 2001) and has been described in several teleost fish (Witeska, 2015). To our knowledge, there is no published information available to assess the nature of this recent health challenge, and no current hematological reference values available for ‘normal’ farmed Atlantic salmon, except in Sandnes and Lie (1988), e.g., Hematocrit 44–49% and Hemoglobin 8.9–10.4 g/dL determined from 140 Atlantic salmon with an average weight of 1.4 ± 0.4 kg. In the light of this, company fish health teams have devised protocols to monitor the extent of this challenge and find ways to identify the mechanisms of anemia, the etiology, and developing preventative strategies. The anemic fish within an affected population has been anecdotally described by fish health managers and veterinarians as lethargic, showing atypical swimming behavior and possible hyperventilation implied by an increased movement of the operculum (pers. comm.). Gross examination of gills showed pallor and pinhead petechiae across the filaments (Fig. 1). Pallor was also observed on internal organs such as the liver following gross postmortem examination, and spun hematocrit/ packed cell volume (here in referred to as Hct) measurements could be <5% in severely anemic fish. Hct categories have been defined by the salmon company health teams based on clinical observations and can be broadly categorized as shown in Table 3.

This review will address (i) an overview of commercially significant and lesser-known health challenges where anemia has been identified in salmonid aquaculture (ii) current and novel methods for anemia assessment (iii) a case study of anemia in Scottish salmon aquaculture.

## 2 Anemia in farmed salmonids

Multiple causative factors can contribute to clinical anemia in fish e.g., viral, bacterial, and parasitic infections, toxins, nutritional deficiencies, and blood loss (Witeska, 2015). This condition reduces the oxygen-carrying capacity in the circulatory system causing tissue hypoxia and consequently results in complications such as reduced growth and elevated mortality rates (Witeska, 2015; Fazio, 2019). Due to the complex interactions of potential causes, it is difficult to distinguish all of the possible factors that may induce clinical anemia under intensive farming conditions, therefore, identifying the etiology and epidemiology of anemia is challenging. The types of anemia can be classified based on changes in red blood cell (RBC) volume (microcytic: smaller; normocytic: normal; macrocytic: larger), Hb concentrations (hypochromic: reduced; normochromic: normal), loss of blood cells (hemolytic or hemorrhagic) and hemopoietic activity (regenerative and non-regenerative) (Tvedten, 2010). The disruption or destruction of hemopoietic tissue leads to non-regenerative anemia associated with causes such as inflammation, nutritional deficiencies, toxins, and tissue damage (Grimes and Fry, 2015). Regenerative anemia, associated with hemorrhaging or hemolysis, can be identified from an increase in immature red blood cells in the circulation (Clauss et al., 2008). A concise evaluation of fish peripheral blood and types of anemia and known causes have been provided in Campbell (2015).

Anemia in salmonid farming has been associated with some of the most commercially significant bacterial and viral infections. Infectious salmon anemia (ISA) is a viral infection of Atlantic salmon caused by an orthomyxovirus (Falk et al., 1997; Rimstad and Mjaaland, 2002). The disease was first registered in Norway in 1984 and was subsequently diagnosed in Scotland, Canada and the Faroe Islands (Lyngstad et al., 2007). ISAV is listed as a notifiable List B disease in Norway and a non-exotic disease under the UK and EU legislation Council Directive 2006/88/EC, due to its virulence and can cause severe financial losses to the industry. If anemia due to ISA is suspected on a farm, a cascade of strict biosecurity and regulatory controls are rapidly put in place and infected stocks isolated and potentially destroyed. In 1998, the Scottish salmon industry experienced its first official outbreak of ISAV, and within 18 months, the viral infection had spread to 11 farms, was suspected in a further 25 farms, and spread over an extensive geographical range (Royal Society of Edinburgh, 2002). In severe clinical cases of ISA, the impact of anemia included Hct values less than 10%, exophthalmia, pale gills, and hemorrhaging and necrosis of various organs (MacLachlan and Dubovi, 2017). Clinical cases continue to be detected in Norway and Canada with variable cumulative mortality rates from ≤5% to ≥90% (Ritchie et al., 2009). The last recorded outbreak in the Scottish salmon industry was in 2009 in the Shetland Isles (Murray et al., 2010). In countries where ISAV is an ongoing challenge, effective vaccines are routinely used in farmed salmon to prevent outbreaks, and molecular testing for the virus gives reliable results allowing rapid confirmation of the diagnosis.

Anemia is also a clinical sign of other known diseases such as bacterial kidney disease (BKD) caused mainly by *Renibacterium salmoninarum* (Delghandi et al., 2020), cold water vibriosis caused by several Vibrio species (Austin, 2006), infectious hematopoietic necrosis virus (IHNV) (St-hilaire et al., 2002) in salmonids and viral hemorrhagic septicemia (VHS) in farmed trout (Raja-Halli et al., 2006; Stone et al., 2008; Dale et al., 2009). Other less known infectious diseases that are associated with anemic symptoms include erythrocytic inclusion body syndrome (EIBS) virus in farmed salmonids (Rodger et al., 1991; Jarp et al., 1996; Rodger and Richards, 1998) and viral erythrocytic necrosis (VEN) in hatchery-reared salmonids (Rohovec and Amandi, 2011). However, it is important to note that molecular testing for most commercially significant viral and bacterial pathogens is commonly available to accurately confirm whether any of these pathogens are involved in a particular case of anemia.

When it comes to sporadic disease events, it can be more diagnostically challenging to confirm the original cause of that outbreak. For example, a case of an anemic outbreak that has been documented in the literature but remains unclear about the etiology was described in 1988. Increased mortality levels were experienced in market-sized fish (2–4 kg) across multiple chinook salmon (*Oncorhynchus tshawytscha*) farms in British Columbia, Canada. Fish farmers termed the condition ‘marine anemia’ due to the pale appearance of the gills accompanied by enlargement of the spleen and kidney and fluid in the body cavity (Kent et al., 1990). This condition has been histologically described as a plasmacytoid leukemia (PL) which showed significant proliferation of plasmacytoid cells (plasmoblasts) in the visceral tissues and occasionally parts of the eye, yet there remained some discrepancy as to what triggered the syndrome. Suggestions included the retrovirus, salmon leukemia virus (SLV) (Eaton and Kent, 1992), BKD (Evelyn, 1993), or an intracellular protozoan parasite (*Enterocytozoon salmonis*) (Elston et al., 1987). Kent and Dawe (1993) determined that neither *R. salmoninarun* nor *E. salmonis* was the causative agents of PL and that a virus or an extremely small organism such as a mycoplasma were most likely responsible for the anemia outbreak. What remains apparent in aquaculture today is that there is a persistent threat to fish health and welfare through the emergence of pathogens driven by multiple factors (Rimstad, 2011; Kibenge, 2019; Shea et al., 2020). Thus, it is challenging to identify causative agents associated with transient or sporadic disease events.

## 3 Current methods to assess anemia in salmon

Hematology is a valuable tool to highlight subtle changes to an individual's health through identifying deviations in the complete blood cell count (CBC). A CBC typically includes the quantification of multiple cellular blood components: RBC, white blood cells (WBC), WBC differential, platelets (thrombocytes in lower vertebrates), hematocrit (Hct), hemoglobin (Hb), and the RBC indices including: mean corpuscular volume (MCV), mean corpuscular hemoglobin (MCH), and mean corpuscular hemoglobin concentration (MCHC).

Fish hematology has gathered momentum for monitoring health in various aquatic sectors (i.e., fisheries science, ornamental pets) and is considered a useful diagnostic method due to its non-destructive nature and the capacity for increasing sample size compared to the gold standard, histopathology (Rough et al., 2005; Snellgrove and Alexander, 2011; Ali et al., 2018; Parrino et al., 2018). Adopting hematology as one of the primary health monitoring services in aquatic diagnostics is currently limited by pre-analytical (sample collection, handling and transport), analytical (instrument performance, established reference values, quality assurance) and post-analytical (data management, robust interpretation) factors (Vap et al., 2012). Nonetheless, efforts to establish reference values and develop best practice guidelines for sample taking, handling, and storage are underway (Hrubec et al., 2000; Clark et al., 2011; Fazio et al., 2013a, Fazio et al., 2013b, Fazio et al., 2017a, Fazio et al., 2017b; Matsche et al., 2014; Dal'Bó et al., 2015; Ivanc et al., 2016; Ciepliński et al., 2019; Duman et al., 2019).

Manual methods for analyzing fish blood parameters tend to be normal practice due to the nature of the blood, which has a high ratio of nucleated RBCs to WBCs, making it challenging for automated analyzers to correctly quantify and categorize cell types (De Keijzer and Van Der Meer, 2002). In veterinary medicine, Hct, RBC count, and Hb are the parameters that would be used during routine examination to determine if an individual is anemic and to what severity (Tvedten, 2010). A detailed explanation of methods used in fish hematology can be referred to in Fazio (2019).

A primary concern in misinterpretation of total WBC counts and the ratio of different classes of WBCs is the time between when a blood sample is taken and when it is analyzed. Faggio et al. (2013) investigated the impacts of storage time on accurately assessing blood parameters and highlighted the significant alterations to Hb, WBC, thrombocytes, MCH, and MCHC in blood analyzed more than 6 h post sampling. These skewed results can have considerable consequences for anemia assessment and interpretation especially as most blood samples can arrive at laboratories for analysis 12–24 h post on-site sampling. Preliminary investigations on WBC changes suggest a 30% reduction in the total WBC count from whole blood assessed within 2 h compared to 12 h from blood withdrawal (unpublished data). Arnold et al. (2014) proposed fixing whole blood in 10% formalin immediately after collection from fish which showed that cell morphology in the fixed sample was maintained for up to 1 month after the blood was taken. On the contrary, our own analysis following the methods provided in Arnold et al. (2014) resulted in lysis of RBCs, compromised cell integrity, shrinking of RBCs and WBCs when compared to unfixed whole blood samples from Atlantic salmon (unpublished data).

## 4 Potential methods to assess anemia in modern day salmon farming

One of the main limitations of using hematology in salmon aquaculture is the lack of well-defined and robust reference values for cultured Atlantic salmon. These shortcomings make it difficult to interpret blood parameters and indices between normal and unhealthy individuals under different farming scenarios. Automated hematology analyzers enable large volumes of samples to be processed for a range of domestic and farmed animals. Fish blood is not currently an available pre-installed option on commercial bench-top hematology analyzers due to nucleation of RBC and morphological similarities of WBC and therefore manual methods are preferred (Arnold et al., 2019). Development of point-of-care (POC) devices and benchtop analyzers are continually emerging, especially in human medicine, but it appears a lack of demand for hematology in fish health diagnostics has hindered development in this area (St John and Price, 2014; Balter et al., 2016, Balter et al., 2018). One exception is the adapted method specific for fish hematology developed by Fazio et al. (2012) using the HeCo V veterinary hematology analyzer (SEAC, Florence, Italy). The impedance analysis system has been developed for fish blood using specific software that can determine the WBC count after subtracting the RBC nuclei that are lysed in reading chambers. The technology has been used in multiple publications from its first development on various fish species (Faggio et al., 2013; Fazio et al., 2013a, Fazio et al., 2013b; Fazio et al., 2015; Fazio et al., 2016; Fazio et al., 2017a, Fazio et al., 2017b). However, we are unaware of the HeCo V veterinary hematology analyzer is commercially available as no results were returned with a simple internet search using the company or instrument name.

To help overcome the issues with blood degradation during transportation and the technical complexity of automated hematology analyzers, cell image analysis software may be an alternative solution for the automation of blood diagnosis. Image analysis has been developed over many years for various cell biology applications but has been typically used to process fluorescent and phase contrast images and limited in the use of light microscopy images (Buggenthin et al., 2013). Digital microscopy, together with cell recognition software, is becoming more established in human hematology, with numerous companies developing a niche market area to compete with traditional manual microscopy of peripheral blood smears (Riedl, 2018). It should be noted that hematological assessment and accuracy for interpretation using this method is dependent on the optimal quality of blood smears made immediately upon blood collection to avoid sample degradation (Arnold et al., 2019). This area has not been developed for fish. However, with advancements made in human hematology, it may be the next technology that can offer rapid diagnostic assessment and high throughput suitable for modern day fish farming (Hutchinson et al., 2005). Point-of-care devices have been tried and tested for fish, and other vertebrate hematology and biochemical endpoints, and a list of species and parameters can be reviewed in Stoot et al. (2014). The validation of POC devices compared to standard laboratory methods varies between devices with various authors reporting levels of acceptable comparability which has been consolidated in Stoot et al. (2014) for measuring hemoglobin and/or hematocrit (Table 2). The results from work undertaken would suggest that the development of a POC device-specific for Atlantic salmon would be more appropriate than attempting to validate technologies that have been manufactured and calibrated for human or mammalian blood. For example, the StatStrip Hb/Hct (Nova Biomedical®, USA) POC device could provide salmon farmers with an anemia screening tool that measures both parameters compared to other handheld devices that calculate Hb from Hct. Although there may not be an economic incentive to primarily develop salmon-specific automated technology, analyzers that could accurately measure blood parameters of nucleated RBCs are more likely transferable to other species such as reptiles, amphibians, and birds, therefore widening the target market.

**Table 2:** Comparison of point-of-care (POC) devices tested on various fish species with laboratory methods adapted from Stoot et al. (2014). NSD (No significant difference).

POC device tested	Species	Blood parameter	Laboratory method	POC compared to the laboratory method	Reference
iStat (EC8+)	Bony fish (*Sebastes melanops, Sebastes mystinus*)	Hb	Spectrophotometry	Significantly lower	(Harrenstien et al., 2005)
HemoCue	Bony fish (*Oncorhynchus nerka, Oncorhynchus tshawytscha, Thunnus orientalis, Scomber japonicus*)	Hct	Spectrophotometry	Significantly higher	(Clark et al., 2008)
BMS Hemoglobinometer	Bony fish (*Salmo salar*)	Hb	Spectrophotometry	NSD	(Iwama et al., 1995)
Ames minilab	Bony fish (*Salmo salar*)	Hb	Spectrophotometry	NSD	(Iwama et al., 1995)
iStat (E3+)	Bony fish (*Albula vulpes*)	Hct	Centrifuge	NSD	(Cooke et al., 2008)
iStat (E3+)	Bony fish (*Fundulus seminolis*)	Hct	Centrifuge	Significantly lower	(DiMaggio et al., 2010)

**Table 3:** Proposed categorization of Hct values and corresponding interpretation to classify Atlantic salmon (*Salmo salar*) population as normal, subclinical, anemic or polycythemia by company veterinarians.

Hct value (%)	Interpretation
> 80	Polycythemia
40–60	Normal
25–40	Sub-clinical
< 25	Anemic

**Table 4:** Percent (%) of active marine farm sites (n = 80) from two major Scottish salmon producers that were reported with suspected or clinical anemia during mid-late summer 2017 to 2019 from several marine regions in Scotland.

Year	West Highlands	Outer Hebrides	Argyll	Clyde
2017	2.5	2.5		
2018	5	6.25		1.25
2019	10	1.25	1.25

**Table 5:** Information provided by two Atlantic salmon farming companies in Scotland (Company A and Company B) describing the observed relationships (*: weak; **: moderate; ***: strong; −: unknown) between positively diagnosed gill disease (identified by company gill scoring practices and histopathology reports) and clinical signs of anemia on 1–13% of total marine farms sites (n = 80) annually during mid-late summer 2017 to 2019.

Year	AGD		PGD		CGD	
	Company A	Company B	Company A	Company B	Company A	Company B
2017	**	–	**	–	***	–
2018	**	*	**	***	***	***
2019	*	*	***	***	**	***

Blood serum biochemical parameters have been used for various health monitoring in fish studies. Some applications include the use of biochemistry to monitor changes in endpoints that represent the analysis of health status linked to pollution effects (Adham et al., 2002; Jaffer et al., 2017), feed trials (Silva-Carrillo et al., 2012; Madibana et al., 2017; Matulić et al., 2020), diagnosis and monitoring of disease (Yousaf and Powell, 2012; Collins et al., 2016), toxicological studies (Öner et al., 2008; Řehulka et al., 2016) and pathophysiology (Amend and Smith, 1975; Kumar et al., 2013). Biomarkers such as ferritin and transferrin, have been used in animal health to examine iron deficiency (Bohn, 2013), but to our knowledge, have not been explored for use in the assessment of anemia in teleost fish. From recent communications, low levels of albumin and globulin have been recorded in farmed Atlantic salmon (*n* = 8) that were identified as clinically anemic from corresponding Hct values of <10% (unpublished data). These biomarkers will help to investigate the cause of anemia such as inflammation, hemorrhaging, or malnutrition (Throop et al., 2004; Werner et al., 2004). Increased levels of bilirubin may indicate too many RBCs being destroyed (hemolysis) and may help to identify hemolytic anemia (Barcellini and Fattizzo, 2015).

Fish immunology and the development of tools and methods to characterize the responding immune system have been explored in response to various pathogens in cultured and wild fish species (Magnadottir and Gudmundsdottir, 1992; Yoshimizu et al., 1992; Kibenge et al., 2002; Okuda et al., 2006; Kim et al., 2008; Estensoro et al., 2012; Castro et al., 2013; Gye and Nishizawa, 2018). Immunoassays – methods for measuring antigen or antibodies – have made it possible to detect if an individual has been infected by a specific antigen or if antibodies are present that can demonstrate exposure to a specific antigen (Lydyard et al., 2000). Immunoassays have been used to study the impact of anemia on the immune systems for patients suffering from iron deficiency (Ekiz et al., 2005; Sadeghian et al., 2010; Hassan et al., 2016) and in domestic/ terrestrial animals, the direct Coombs test is commonly used for detecting immune-mediated hemolytic anemia (IMHA) (MacNeill et al., 2019). Currently, there does not appear to be adequate antibody testing for the detection of IMHA in salmon aquaculture due to a lack of fish-specific immunoassays.

## 5 Anemia in Scottish salmon farming: A case study

Reports of an unexplained reduction in growth potential and mortality cases from five out of eight farming companies across Scotland were described from mid-late summer during 2017 to 2019. The principal symptoms reported by veterinarians and biologists in the field included gill pallor and micro-bleeding presenting on the gills (Fig. 1) and some internal organs. Analysis of blood samples taken from moribund and live fish indicated critically low hematocrit values (< 20%), confirming the presence of anemia. In contrast to the ISA outbreak in 1998 there was no confirmed link to ISAV. Due to the lack of reference values for farmed Atlantic salmon and non-specific histological findings, it remains challenging to fully understand the primary etiology of the blood disorder and for mitigation measures to be put in place.

**Fig. 2:**
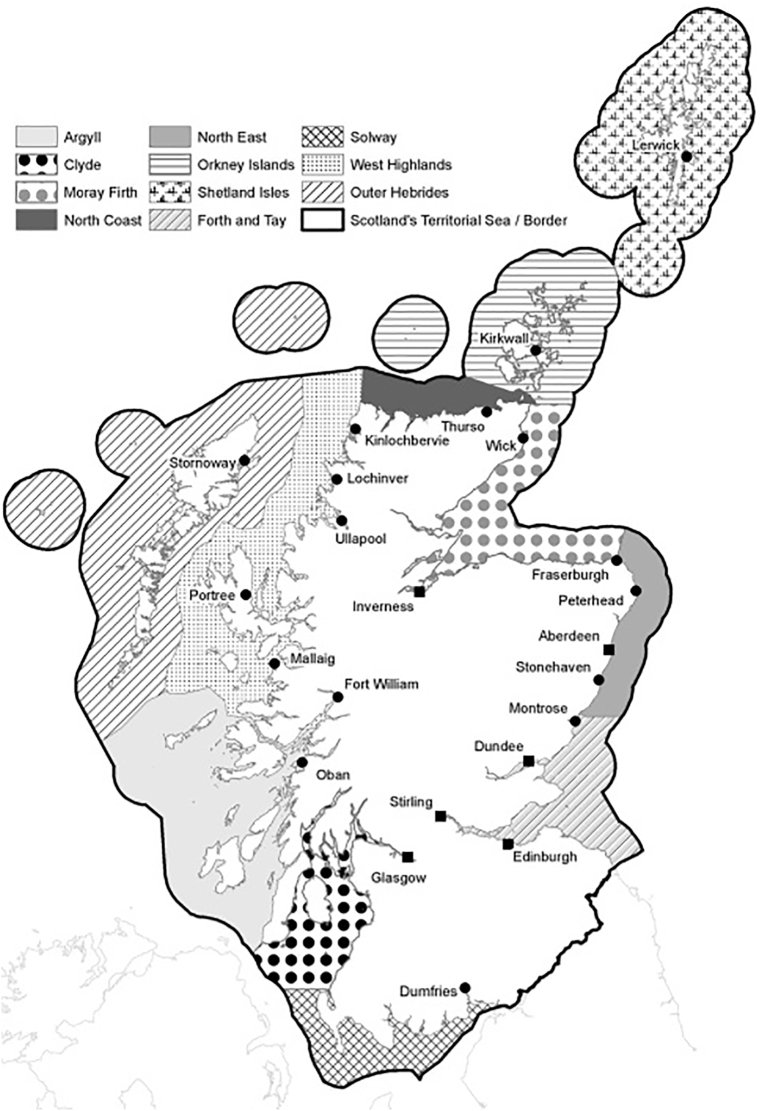
Results from questionnaires completed by two Scottish salmon farming companies that suspected or identified clinical anemia on 1–13% of active marine farms (*n* = 80) annually during mid-late summer 2017–2019 in the marine regions of West Highlands, Outer Hebrides, Argyll and Clyde. The capital cities (squares) and the major towns (circles) in Scotland are provided (Marine Scotland, Crown copyright). Further details on precise locations of the farms are not provided.

Industry-based veterinarian and health teams from two out of the five affected salmon farming companies (Company A and Company B) completed questionnaires (Fig. S1) that covered the initial outbreaks in mid-late summer 2017 until 2019 (excludes any subsequent years). The total number of samples during this period for Company A and Company B included Hct (n = 104,339), blood smears (*n* = 695), gross examination of gills and fish condition (*n* = 104,339). The results indicated a wide geographical distribution of sub-clinical and clinical cases of anemia throughout the Scottish marine regions of Clyde, Argyll, West Highlands, and Outer Hebrides (Fig. 2). Cases of anemia in <5 sites were confirmed in the Orkney Islands and the Shetland Isles (pers. comm.) and are not included in the current case study. From the total number of active marine sites (*n* = 80) in Company A and Company B during mid-late summer 2017 to 2019, it is estimated that between 1 and 13% were affected by anemia annually (Table 4). Hct values between 25 and 35% were recorded from clinical cases, with some individuals measuring with Hct < 10%. One of the few consistent characteristics of clinical anemia in the salmon farms was the rapid decline in Hct values and the onset of mortality following an initial outbreak in one or two pens, typically between 7 and 15 days.

The industry questionnaires indicated that the incidence of anemia may be correlated with the deterioration in gill health associated with one or multiple common gill health challenges: amoebic gill disease (AGD), proliferative gill disease (PGD), or complex gill disease (CGD). AGD can have significant health and welfare implications and cause economic loss with reports of up to 82% mortalities (Steinum et al., 2008; Rodger, 2013; Hvas et al., 2017). PGD is a syndrome mostly associated with gross lesions in the salmon gills, and CGD can often include inflammation of the gill filaments, excess mucus development, shortened gill filaments, petechial hemorrhages, and paleness to the filaments (Herrero et al., 2018). Company A and Company B were asked to rank the relationship between fish with anemia (based on Hct values <35%) and gill disease from corresponding industry gill scores and histopathology (Mitchell et al., 2012) reports as weak, moderate, or strong for each year that anemia was detected (Table 5).

The results showed that the relationship tended to be strongest when populations showed pathologies for CGD from routine histopathology. Gill disease is a considerable health challenge experienced in modern day farming during the marine stage of growth (Rozas-Serri, 2019). However, defining the precise etiology of gill disease has remained ambiguous due to the numerous infectious agents (Atlantic salmon paramyxovirus-ASPV, Salmon gill poxvirus-SGPV, *Tenacibaculum maritimus, Piscichlamydia salmones, Candidatus Clavochlamydia salmonis, N. perurans, Loma salmonae, Ichthyobodo* spp*., Trichodina* sp.) and non-infectious agents (phytoplankton and zooplankton) that could be present on the gills (Matthews et al., 2013). However, inconsistencies remain between farms as not all stocks that exhibit PGD/ CGD develop severe anemia and can recover quickly. These discrepancies between farms indicate the potential for further investigation into the presence of other etiologies that may impair the blood coagulation system.

Observations made by Company A based on blood smears (n = 695) and Hct values (*n* = 96,000) suggest that in most cases it is regenerative anemia, resulting from loss of RBC (hemorrhage) or destruction of RBC (hemolysis). Examination of blood smears revealed no obvious evidence of hemolysis as there was no intracellular hemosiderin within neutrophils or monocytes. A small number of histological results (*n* = 5) did not show hemolytic anemia due to the lack of melanomacrophage centers with high hemosiderin content (Agius and Roberts, 2003; Steinel and Bolnick, 2017), leading to the suggestion that the anemia is most likely linked to the microhemorrhages identified on the gills and liver. What remains unknown is why some farms suffer acute anemia while others develop chronic anemia that can lead to reduced growth. There is also a dearth of prognostic indicators to forecast whether a population is likely to make a full recovery or whether the severity of the anemia and risk of mortality will escalate.

## 6 Conclusions and recommended research

The occurrence of anemia in farmed Atlantic salmon in Scottish aquaculture has left fish health teams puzzled as it appeared to emerge rapidly with the minimal diagnostic capability to identify the precise cause or causes. Following the observation of episodes of anemia in fish stocks, an increased focus has emerged for the use of routine blood samples to screen, diagnose and monitor health in fish stocks as a non-lethal approach to health management and to optimize welfare. Through this study, progress has been made to identify that fish are experiencing regenerative anemia that is most likely linked to blood loss via the fish gills during periods of gill health challenges. However, what remains to be understood is why some farms do not recover from these anemic events, and others do?

To establish hematology as a diagnostic tool for continuous health monitoring in salmon aquaculture, there needs to be a robust set of reference values specifically for farm-raised Atlantic salmon (and other farmed finfish) to enable accurate interpretation of blood parameters as has been established for many species in veterinary medicine. Due to the complex nature of intensive farming of salmonids, in addition to the multiple factors that influence hematological values, it is an arduous task to establish ‘normal’ reference ranges using traditional manual methods for a CBC. To enable the development of a representative reference dataset, it would be beneficial to develop automated systems to handle large sample volumes for rapid screening, whether that be from laboratory-based hematology analyzers or handheld devices for use on-site. For a holistic approach to health management and complementary methods for hematology examination, the use of clinical chemistry, with endpoints such as ferritin, bilirubin, albumin, and globulin, would aid in the identification of the mechanisms and provide another non-invasive method for health monitoring in aquaculture.

## Declaration of interests

The authors declare that they have no known competing financial interests or personal relationships that could have appeared to influence the work reported in this paper.

## Declaration of Competing Interest

None.

## Appendix A Supplementary data

Supplementary Fig. S1 Questionnaire template provided to two major Scottish farmed Atlantic salmon producers to establish information related to the anemia outbreaks. Image 1
