# Supplementary material for: Anemia in salmon aquaculture: Scotland as a case study
Source: Aquaculture. 2022 Jan 15;546:737313. doi: 10.1016/j.aquaculture.2021.737313 (PMC8547259; doi:10.1016/j.aquaculture.2021.737313)
Supplement: Supplementary Fig. S1 — Questionnaire template provided to two major Scottish farmed Atlantic salmon producers to establish information related to the anemia outbreaks. [file mmc1.pdf]

UWS UNIVERSITY OF THE WEST *of* SCOTLAND

Information gathering exercise Nov-19

Anaemia in farmed Atlantic salmon: occurrence, distribution & causes

Thank you for your participation in this brainstorming survey, which aims to capture an industry perspective of occurrence, distribution and possible causes relating to anaemic events in farmed Atlantic salmon since 2017.

Please answer the following questions with as much detail as possible and please note that we do not require any company-sensitive information.

From your experience:

Q1. **Occurrence**. Has the incidence level of anaemia *increased/reduced/same* since first detected? Please write your answer under each sub-heading.
Month/Year first detected<br><br>2017<br><br>2018<br><br>2019

Q2. **Distribution**. Has the distribution of anaemia *increased/decreased/same* over a geographical scale since first detected? Please write your answer under each sub-heading. ***N.B. If you prefer, please mark distribution on map provided on last page of survey.***
Location first observed<br><br>2017 observations<br><br>2018 observations<br><br>2019 observations

Q3. **Type**. Has the presentation of anaemic fish changed between years? Please tick boxes that apply to anaemic characteristics experienced each year or note any other significant observations.

2017						
Microhaemorrhage on gills	Palor of the gills	Oxidised liver	Palor of other organs	Palor of body wall	Bleeding from gills	No bleeding from gills
Other						

2018						
Microhaemorrhage on gills	Palor of the gills	Oxidised liver	Palor of other organs	Palor of body wall	Bleeding from gills	No bleeding from gills
Other						

2019						
Microhaemorrhage on gills	Palor of the gills	Oxidised liver	Palor of other organs	Palor of body wall	Bleeding from gills	No bleeding from gills
Other						

Any further comments:

Q4. **Causes**. Would you say anaemia is *<u>more/less</u>* related to poor gill health? Please best describe the correlation (i.e. weak, moderate, strong) between the following:

<u>Anaemia and AGD</u>

2017 -
2018 -
2019 -

<u>Anaemia and PGD</u>

2017 -
2018 -
2019 -

<u>Anaemia and CGD</u>

2017 -
2018 -
2019 -

Q5. **Causes**. Anaemia in salmon may be induced by various factors. Under the sub-headings, please share some ideas as to what you believe is triggering anaemia in farmed salmon populations.

Toxic chemicals

Viral and bacterial infections

External or internal parasite

Nutritional deficiencies

Starvation

Bleeding

Other

Q6. **Causes**. If anaemia was detected on a farm, were there any links that can be made to the following sub-headings. Please provide details.

Stock type

Diet

Cage location

Stocking density

Treatments

Cleaner fish

Lice skirts

Hydroids

Other (any ideas may be of extreme value)

Q7. Has there been any indication from parent companies that anaemia has been a significant health challenge in other farming countries i.e. Norway, Canada, Faroe Islands etc.

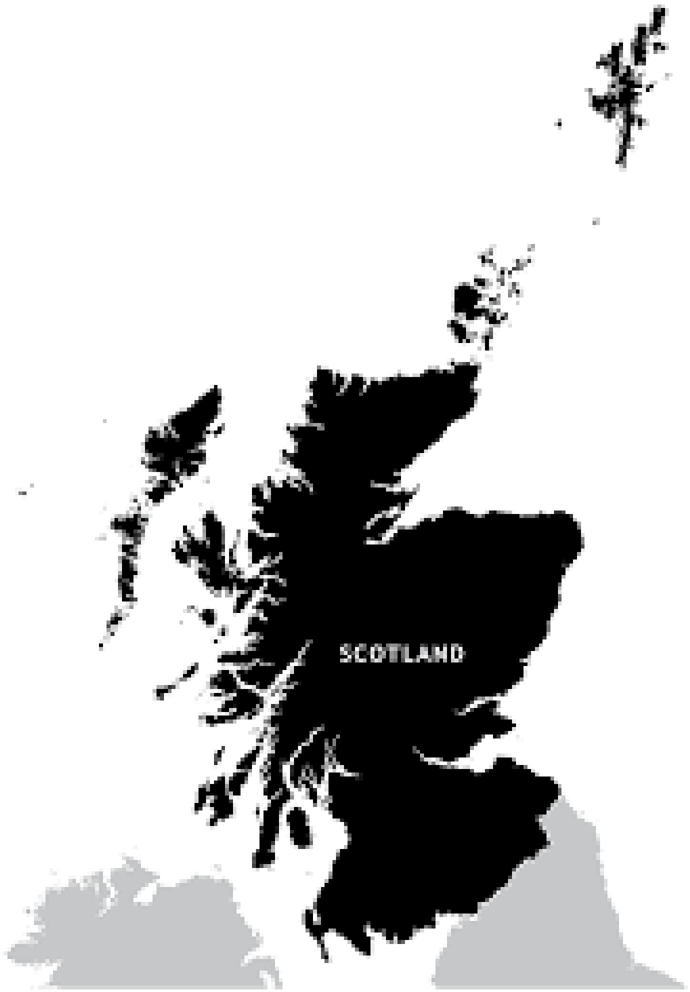

Q2 Cont’d. Please mark map to show distribution of anaemic events. If distribution changes per year, please use different coloured markers to represent and note colour = year
